# Targeted Peptide Nanofiber-Loaded Stomatocytes for Combined Photodynamic and Photothermal Therapy

**DOI:** 10.1021/acsami.5c06448

**Published:** 2025-06-03

**Authors:** Yuechi Liu, Duc H.T. Le, Gokhan Yilmaz, Lars Paffen, Shukun Li, Alexander B. Cook, Tania Patino Padial, Loai Abdelmohsen, C. Remzi Becer, Bingbing Sun, Jan C.M. van Hest

**Affiliations:** † Bio-Organic Chemistry, 3169 Institute for Complex Molecular Systems, Eindhoven University of Technology, P.O. Box 513, 5600 MB, Eindhoven, Helix, The Netherlands; ‡ Department of Chemistry, 2707 University of Warwick, Coventry CV4 7AL, U.K.

**Keywords:** peptide conjugates, nanofiber, stomatocytes, photodynamic therapy, glycopolymer

## Abstract

Nanotechnology-based photodynamic (PDT) and photothermal (PTT) therapies offer precise treatment by generating reactive oxygen species (ROS) and localized hyperthermia. However, photosensitizer nanodrugs suffer from rapid degradation and limited tumor targeting. To overcome these challenges, we have developed a dual-nanoparticle drug delivery platform based on bowl-shaped polymer vesicles, which were loaded in the protective nanocavity with nanofibers composed of a histidine dipeptide-photosensitizer conjugate. The stomatocytes were surface modified with mannose glycopolymers to induce targeting capacity. This dual nanoparticle platform exhibited enhanced tumor imaging, improved targeting, and increased cellular uptake in tumor cells and 3D tumor spheroids. Under 660 nm laser irradiation, it demonstrated excellent PTT and PDT performance, with significant ROS generation and temperature elevation. This advanced nanomedicine platform, therefore, offers a promising approach for precision cancer therapy.

## Graphical abstract

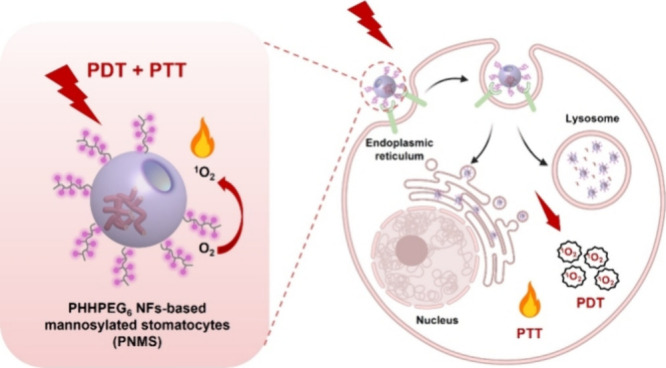

## Introduction

Cancer remains one of the most challenging diseases to treat, with conventional therapies often limited by poor specificity, systemic toxicity, and resistance mechanisms.^1–3^ In recent years, advances in nanotechnology, particularly photodynamic therapy (PDT) and photothermal therapy (PTT), have emerged as promising strategies for precise cancer treatment.^4,5^ These therapies leverage the generation of reactive oxygen species (ROS) and localized hyperthermia to induce cancer cell death, improving the precision and efficiency of tumor eradication while minimizing damage to healthy tissues.^6,7^ A distinctive hallmark of PDT/PTT is their dual selectivity and spatiotemporal control, which rely on the selective accumulation of the photosensitizers in tumor tissues and the activation of therapeutic agents only in targeted areas upon light exposure. This allows precise control over treatment location and duration.^8^ Therefore, designing nanoparticles with photosensitizing capabilities that can be selectively activated within tumor tissue is highly desirable for improving therapeutic efficacy.^9,10^ A class of PDT/PTT nanodrugs that have gained significant attention recently are peptide-based carrier systems.^11,12^ These systems are typically formed through the conjugation of a photosensitizer with a peptide building block, leading to the self-assembly of nanostructures. Their physicochemical properties and photophysical performance can be optimized to suit specific therapeutic applications, such as responding to tumor stimuli, enabling controlled and on-demand drug release, thereby increasing treatment precision.^13,14^

Nonetheless, peptide-based photosensitizer nanodrugs face several challenges. First, they can be degraded rapidly due to enzymatic hydrolysis,^15^ pH effects,^16^ and early elimination by the immune system, resulting in a short half-life in circulation.^17^ Second, nonspecific photosensitizer nanodrugs suffer from insufficient tumor targeting, limited tissue penetration, inefficient cellular uptake and reduced therapeutic efficacy. Because of these limitations, the strategic design and engineering of optimized photosensitizer nanodrugs remain an active area of research. One promising often overlooked strategy is the hierarchical assembly approach, in which a photosensitizer nanodrug is encapsulated inside a carrier system. The biological activity of the therapeutic agent is then unperturbed, while the carrier provides protection and potential targeting capacity.

In light of this, we rationally designed a dual-nanoparticle polymeric drug delivery platform, PHHPEG_6_ NFs-based mannosylated stomatocytes (PNMS) for tumor targeting and combined PDT and PTT (Scheme 1A). Based on our previous research, we selected as therapeutic nanodrug a porphyrin-peptide construct, which was composed of a porphyrin modified with two histidine dipeptides conjugated with a short PEG chain (Scheme 1B, PHHPEG_6_). We demonstrated that this building block self-assembles into nanofibers that exhibit pH-responsive aggregation at pH levels of pH 6.5 and 5.0.^12^ These nanofibers exhibit noteworthy photothermal, photodynamic and fluorescence characteristics upon laser irradiation. As a protective carrier for these nanofibers we selected bowl-shaped polymer vesicles, or stomatocytes, composed of block copolymers of poly­(ethylene glycol) and poly­(D,L-lactic acid) (PEG–PDDLA). The inclusion of PEG in the copolymer increases nanoparticle stability, prevents rapid release, and reduces early elimination by the immune system,^18^ while improving retention in tissues and draining lymph nodes.^19,20^ By modifying the surface of the stomatocytes with mannose glycopolymers, targeting of mannose receptors which are expressed on for example Hep G2 cells could be achieved.^21–25^ Based on the local concentration of nanofibers and targeting efficacy of the mannosylated stomatocytes, this platform improves the PDT/PTT therapeutic effect by effectively concentrating the therapeutic agents in target cells or tissues.

**Scheme 1:**
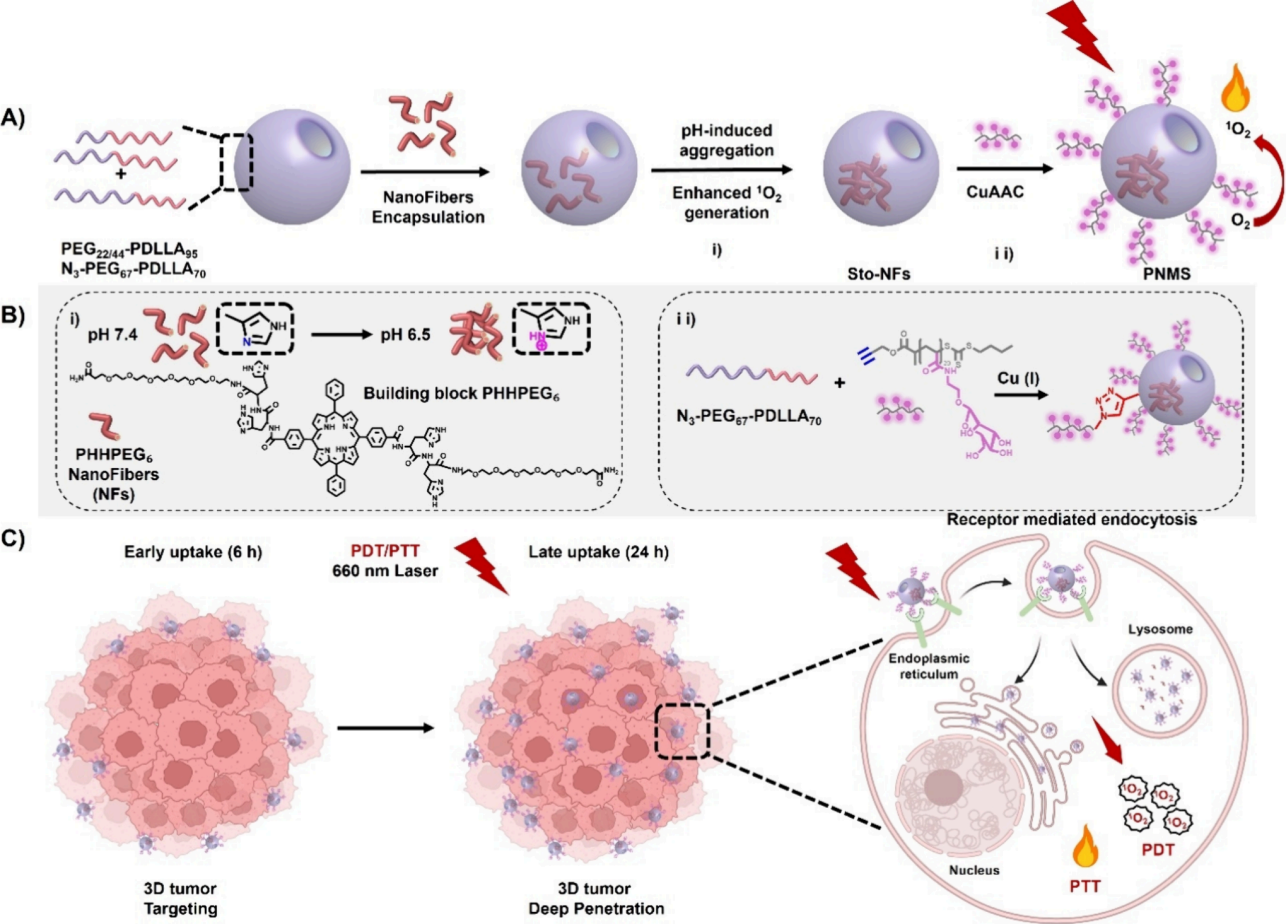
Schematic of pH-Responsive Porphyrin-Peptide Nanofibers Encapsulated in Mannosylated Stomatocytes for Enhanced Photodynamic and Photothermal Therapy^a^

a (A) Stomatocytes are co-assembled from PEG–PDLLA block copolymers; 5% of the PEG chains are azide modified. The nanofibers are encapsulated within the cavity of the stomatocytes and subsequently aggregated by lowering the pH to 6.5, nanofiber-loaded stomatocytes (Sto-NFs)-lack mannose glycopolymer on their surface. Mannose glycopolymers are later attached to the stomatocyte surface through click chemistry, PHHPEG_6_ NFs-based mannosylated stomatocytes (PNMS). (B) (i) Schematic representation of the pH-induced aggregation of the peptide nanofibers (NFs). (ii) Mannose glycopolymer conjugated to stomatocytes via CuAAC click chemistry. (C) Schematic depicting how fiber loaded mannosylated stomatocytes could enhance targeting ability, deep penetration, and effective PDT/PTT therapy in 3D tumor spheroids. Receptor-mediated endocytosis facilitates their entry into Hep G2 cells. Schematic illustrations of tumor and cell endocytosis were created with BioRender.com.

Compared with previous studies of stomatocyte-based or peptide-nanofiber systems,^12,26^ our PNMS platform incorporates several notable advancements that contribute to its enhanced therapeutic performance. First, a novel design feature of our system is the integration of mannose-functionalized glycopolymers on the surface of stomatocytes, enabling active targeting of mannose receptor-expressing cancer cells such as Hep G2.^21–25^ This targeting capability significantly enhances cellular uptake and treatment selectivity, which was largely absent in earlier nontargeted stomatocyte systems. Second, our system achieves improved intracellular delivery by encapsulating pH-responsive porphyrin-peptide nanofibers within the stomatocytes. This architecture increases colloidal stability, protects the nanofibers from premature degradation to achieve effective transport and protection, and concentrates them in the cavity of the stomatocytes—features that previous nanofiber-based platforms did not address. Finally, these innovations result in high efficacy: while free nanofibers alone demonstrated around 80% cell killing in earlier studies,^12^ our PNMS system achieved a higher cytotoxic effect (up to 87%) even at lower nanofiber concentrations, underscoring the benefit of combining structural confinement with active targeting for synergistic PDT/PTT cancer therapy. Based on the local concentration of nanofibers and targeting efficacy of the mannosylated stomatocytes, this platform improves the PDT/PTT therapeutic effect by effectively concentrating the therapeutic agents in target cells or tissues. This dual nanodrug system would therefore retain the benefits, while alleviating some of the drawbacks of peptide-based nanodrugs, and provides a promising strategy for developing next-generation smart nanomedicines for precision cancer therapy.

For the construction of the stomatocyte carrier we synthesized a series of functional polymers, namely PEG_44_-PDLLA_95_, PEG_22_-PDLLA_95_, and N_3_-PEG_67_-PDLLA_70_, via ring-opening polymerization (ROP). Additionally, the pH responsive PEGylated porphyrin-peptide conjugate, PHHPEG_6_, was synthesized by solid phase peptide synthesis. This conjugate was obtained by derivatizing a porphyrin with histidine dipeptides and short PEG chains. Nanofiber formation was achieved by self-assembly. Furthermore, an alkyne-functionalized mannose glycopolymer was synthesized through reversible addition–fragmentation chain transfer (RAFT) polymerization. The resulting block copolymers, peptides and glycopolymers were consistent with those described in our previous work.^12,26,27^ Next, we constructed PHHPEG_6_-loaded mannosylated stomatocytes, as illustrated in Scheme 1. First, the fabrication process commenced with the self-assembly of spherical polymersomes from block copolymers of PEG_44_-PDLLA_95_, PEG_22_-PDLLA_95_, and N_3_–PEG_67_-PDLLA_70_ (10:7:3 w/w/w), utilizing the conventional solvent switch methodology. Gradual removal of the organic solvent via dialysis facilitated an osmotically driven morphological transition, resulting in the formation of stomatocytes. The distinctive dual-compartment structure of the azido-functionalized stomatocytes was demonstrated using cryogenic transmission electron microscopy (cryo-TEM) (Figure 1A) and scanning electron microscopy (SEM) (Figure S1).

**Figure 1:**
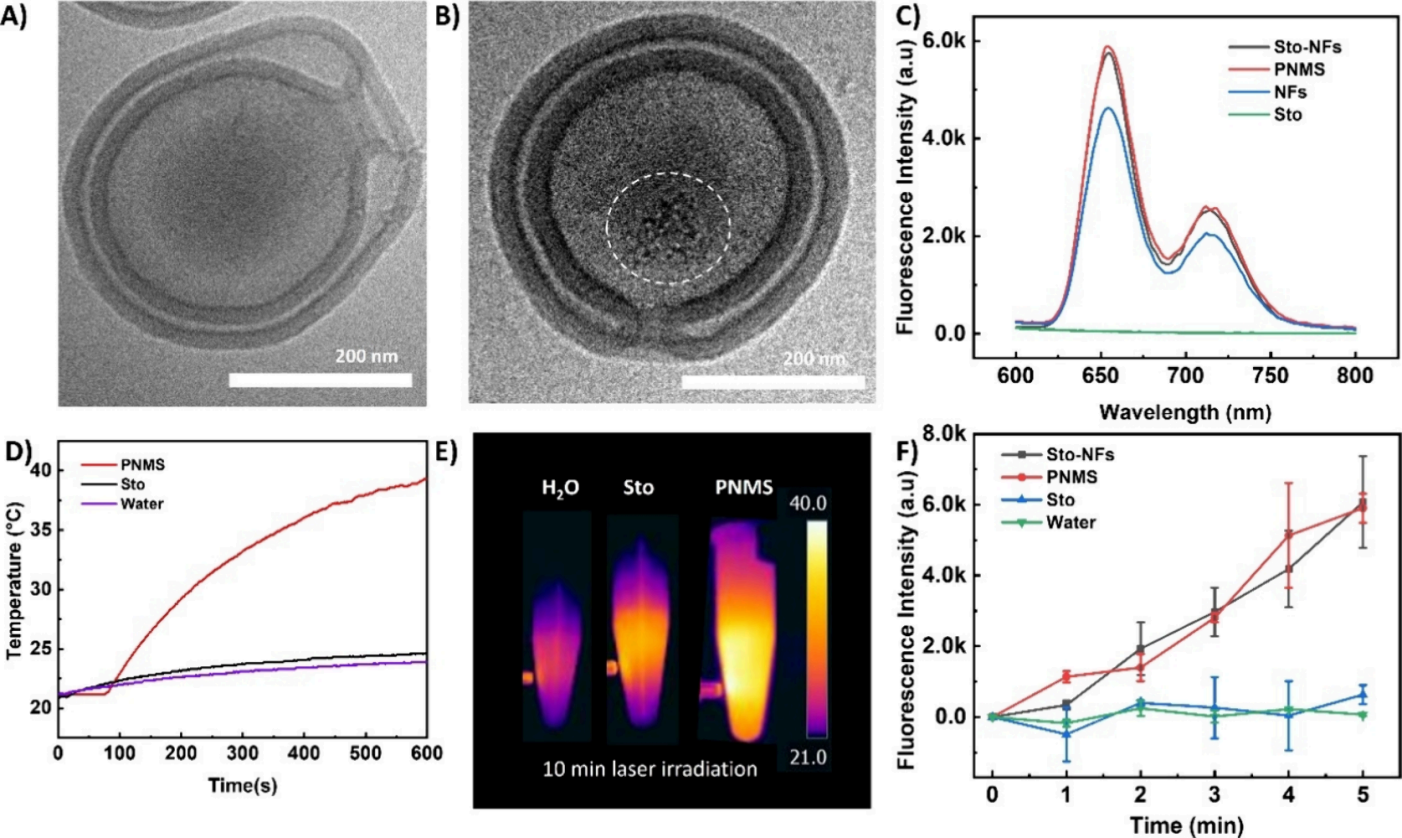
(A) Morphological characterization of empty stomatocytes using cryo-TEM, scale bar = 200 nm. (B) Cryo-TEM image revealing the structure of the nanofiber-loaded mannosylated stomatocytes (PNMS), scale bar = 200 nm. The white dashed circle refers to the aggregated PHHPEG_6_ nanofibers inside stomatocytes. (C) Fluorescence intensity of nanofiber-loaded mannosylated stomatocytes (PNMS), nanofiber-loaded stomatocytes (Sto-NFs), nanofibers (NFs), stomatocytes (Sto). (D) Temperature variation and (E) photothermal images of H_2_O, stomatocytes, and PNMS solutions under irradiation of a 660 nm laser at a power density of 0.2 W/cm^2^. (F) Increased emission of SOSG at 504 nm as a function of time by irradiating PNMS, Sto-NFs, stomatocytes, and Milli-Q water.

We then investigated the inclusion of the PHHPEG_6_ nanofibers within the stomatocyte nanocavity. The encapsulation process involved overnight mixing of the nanofibers with stomatocytes, followed by three successive washing steps to remove unencapsulated fibers. To improve fiber retention in the cavity, they were aggregated at pH 6.5. Specifically, the imidazole groups of the histidine residues (p*K*_a_ ≈ 6.0) facilitated aggregation under slightly acidic conditions. To evaluate the colloidal stability of our dual-nanoparticle platform (PNMS), including sto-NFs and the stomatocyte carriers, we performed dynamic light scattering (DLS) measurements in PBS under various pH conditions (5.0, 6.5, and 7.4), mimicking the pH values of intracellular lysosomes, tumor tissue and healthy tissue. The results demonstrated that all components maintained their hydrodynamic size and low polydispersity, indicating good short-term stability under physiological conditions. At pH conditions 5.0 and 6.5 an increase in size after 24 h was observed, suggesting that the nanostructures degraded, whereas no significant size change was found at pH 7.4 (Figure S2). This is partially related to the fact that the stomatocytes are formed from biodegradable block copolymers (PEG–PDLLA), which are known to undergo degradation under acidic conditions such as pH 5.0.^28^ In addition, the nanofibers remained stable and aggregated under PBS conditions at pH 5.0 and 6.5, while at pH 7.4, they were stable and well-dispersed. As shown in Figure 1B, Cryo-TEM analysis revealed the presence of the nanofibers within the internal cavity of the stomatocytes. Furthermore, stomatocytes and mannosylated stomatocytes loaded with nanofibers exhibited characteristic fluorescence similar to that of the free nanofibers, confirming the successful loading (Figure 1C). These results demonstrate the loading of the nanofibers into the stomatocytes, achieving a loading efficiency of 7.7% (Table S1). Finally, the azido stomatocytes were conjugated with alkyne-functionalized mannose glycopolymer via CuAAC click chemistry, yielding the final nanoplatform.^29^

The PTT potential of the fiber-loaded stomatocytes was evaluated by monitoring real-time temperature changes of their solutions under 660 nm laser irradiation (0.2 W/cm^2^) for 10 min. As displayed in Figure 1D, a significant temperature increase was observed in the nanoparticle suspension, reaching 39.8 °C after 10 min of irradiation. In contrast, control groups containing only Milli-Q water and empty stomatocytes exhibited minimal temperature changes under identical conditions. Thermographic imaging further confirmed the photothermal performance, with the fiber-loaded nanoparticles showing a pronounced temperature increase compared to the control groups (Figures 1E, S3, and S4). These results confirmed the excellent photothermal capability of this platform. To evaluate the singlet oxygen (^1^O_2_) generation efficiency of the fiber-loaded stomatocytes, singlet oxygen sensor green (SOSG) was employed as ^1^O_2_ indicator. As shown in Figure 1F, both particles displayed comparable ^1^O_2_ generation capabilities, with the fluorescence intensity of SOSG endoperoxide (SOSG-EP) gradually increasing over time under 660 nm laser irradiation (0.2 W/cm^2^). This fluorescence enhancement confirms the successful generation of ^1^O_2_, as SOSG specifically reacts with singlet oxygen to produce a fluorescent signal. In contrast, the controls showed negligible changes in ^1^O_2_ generation, further demonstrating the effective photosensitizing properties of the fiber-loaded stomatocytes.

Subsequently, we conjugated azido-stomatocytes with alkyne-modified mannose glycopolymers via CuAAC. The presence of the mannose glycopolymer on the modified stomatocytes was investigated by evaluating their binding affinity to mannose-binding lectin (MBL) using surface plasmon resonance (SPR) analysis.^30^ As depicted in Figure 2A,B, binding effectiveness to MBL was assessed using various concentrations of nanofiber-loaded mannosylated stomatocytes (PNMS) and unloaded mannosylated stomatocytes, ranging from 8 to 0.5 μM. Both stomatocytes exhibited significant binding to MBL at all concentrations, while unmodified stomatocytes, both with and without nanofibers, showed no detectable binding to MBL as negative controls (Figure 2C and S5). This analysis confirmed the successful conjugation of the mannose glycopolymer to the stomatocytes, validating their effective functionalization. The kinetic parameters of the interactions, including the association rate constant (*k*_a_), dissociation rate constant (*k*_d_), and equilibrium dissociation constant (*K*_D_), were determined from fitting of the experimental SPR curves with a 1:1 Langmuir binding model (Table S2). The low dissociation rate constants (*k*_d_ = 1.05 and 5.56 × 10^–6^ s^–1^) observed for both mannosylated stomatocytes and PNMS suggest that the mannose units predominantly remain bound or rapidly rebind upon release, rather than fully dissociating and is in line with earlier observations.^28^ Overall, both mannosylated stomatocytes and PNMS exhibited comparable binding profiles and strong affinity for MBL, indicating that the presence of NFs within the stomatocyte cavity has minimal to negligible influence on their functionalization.

**Figure 2:**
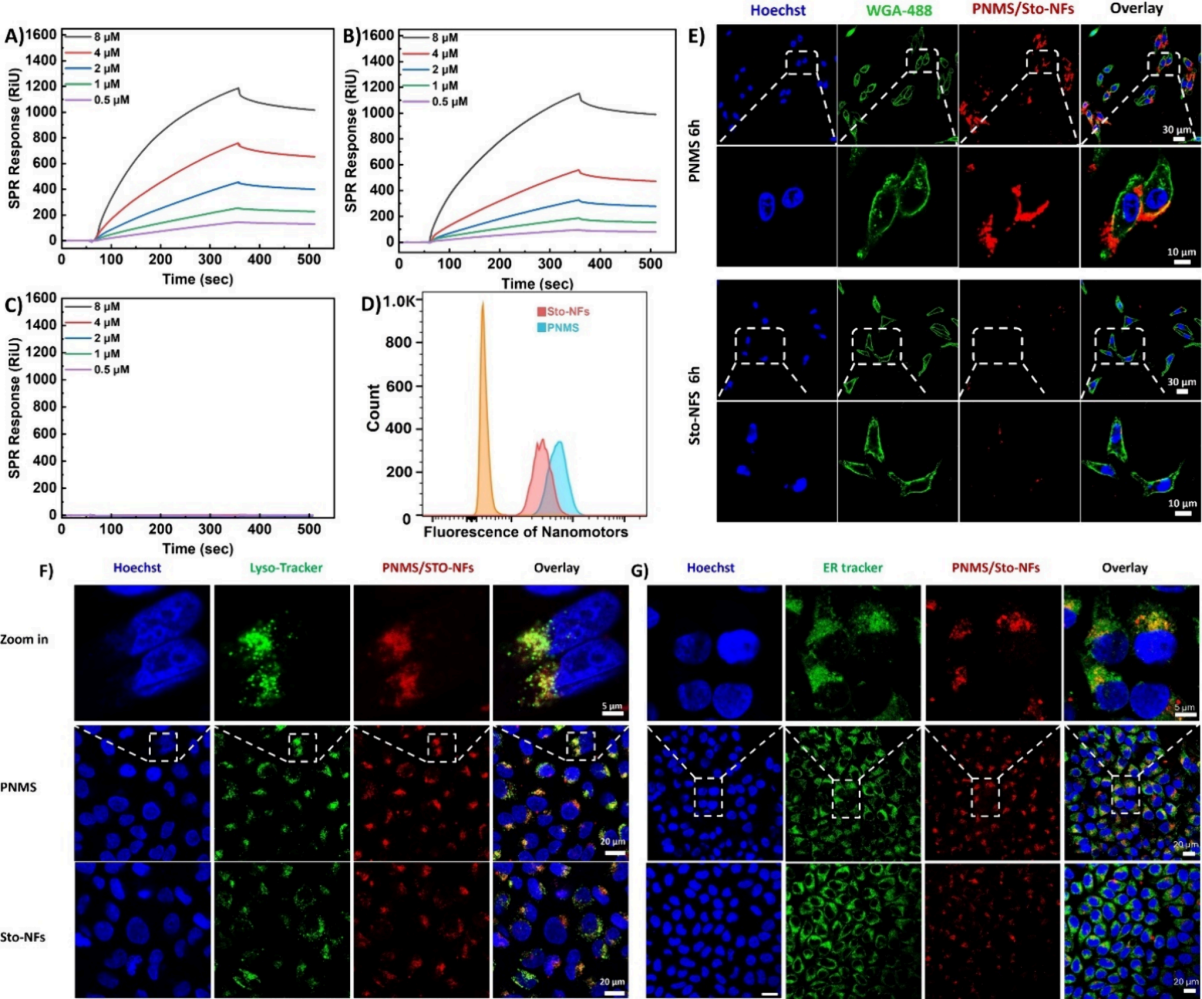
(A) Surface plasmon resonance (SPR) analysis illustrates the binding of PNMS with Mannose Binding Lectin (MBL). (B) SPR analysis demonstrates the binding of mannosylated stomatocytes with MBL. (C) SPR analysis of the binding of Sto-NFs with MBL. (D) Fluorescence histograms of Hep G2 cells incubated with PNMS and Sto-NFs at 6 h. (E) CLSM images indicating the nuclei (stained with Hoechst), cell membrane (Wheat Germ Agglutinin (WGA-488)), and PNMS and Sto-NFs (red), including the overlay. The scale bars are 30 and 10 μm. (F) CLSM images indicating the nuclei (stained with Hoechst), lysosomes (LysoTracker Green), and PNMS and Sto-NFs (red), including the overlay. The scale bars are 20 and 5 μm. (G) CLSM images indicating the nuclei (stained with Hoechst), endoplasmic reticulum (ER) Tracker green, and PNMS and Sto-NFs (red), including the overlay. The scale bars are 20 and 5 μm.

After confirming the successful conjugation of the mannose glycopolymer to the stomatocytes, we next investigated the targeting ability of PNMS in vitro. To assess the cell targeting efficiency of our nanoparticles, Hep G2 cells were incubated with these particles and fiber-loaded stomatocytes without mannose glycopolymer displayed on the surface (Sto-NFs) for 6 h, followed by fluorescence activated cell sorting (FACS) analysis. As shown in Figure 2D, cells treated with PNMS exhibited significantly higher fluorescence compared to those treated with Sto-NFs, indicating that the mannose glycopolymer enhanced cellular uptake efficiency.

The targeting efficiency of the PNMS and Sto-NFs in Hep G2 cells, following 6 h of nanoparticle treatment, was also analyzed using confocal laser scanning microscopy (CLSM). Wheat germ agglutinin (WGA-488) was used to mark the cell membrane, while PHHPEG_6_ nanofibers displayed a red fluorescent signal, enabling visualization of their cellular uptake and intracellular localization. As depicted in Figure 2E, CLSM images and their magnified images showed that PNMS localized on the cell membrane and exhibited significantly higher fluorescence compared to Sto-NFs. These results confirm that mannosylation enhanced cellular fluorescence accumulation through specific binding with Hep G2 cells, suggesting more efficient internalization. This observation closely aligns with the FACS analysis, supporting the conclusion that the enhanced targeting efficiency is likely due to the interaction between the mannose glycopolymer and mannose receptors expressed on the Hep G2 cell membrane.^22–25^

Next, the cellular uptake and intracellular colocalization of PNMS and Sto-NFs were investigated after incubating with cells for 24 h. Hep G2 cells were stained with LysoTracker Green and endoplasmic reticulum (ER) Tracker green markers. In Figure 2F,G, the CLSM images and their magnified views revealed the enhanced cellular uptake of PNMS compared to Sto-NFs at 24 h, with the red fluorescence from the nanoparticles partially colocalized with the green fluorescence in the lysosome and endoplasmic reticulum. The Pearson correlation coefficients for cells were 0.59 and 0.45 for lysosomes and the endoplasmic reticulum, respectively, indicating significant colocalization and suggesting that the nanoparticles mainly localize to these organelles. It is generally accepted that mitochondria- and endoplasmic reticulum-localizing photosensitizers trigger apoptosis via cytochrome C release,^31,32^ while lysosomal photosensitizers induce protease release.^33^ Moreover, the time-dependent localization of nanoparticles in lysosome was also evaluated at 12 and 18 h using CLSM. Figures S6 and 2F,G show that the red fluorescent signals of nanoparticles intensified with prolonged incubation time. These results confirm that PNMS exhibit enhanced cellular uptake in vitro and distributed predominantly to lysosomes and the endoplasmic reticulum. We also investigated the release behavior of the peptide nanofibers from PNMS after incubation with Hep G2 cells using CLSM. For this purpose the stomatocytes were labeled with FITC. The partial colocalization of the fluorescent signals originating from the nanofibers and stomatocytes revealed that some nanofibers remained encapsulated within the stomatocytes, while others were released (Figure S7). This observation provides valuable insights into the release dynamics of the nanofibers, which aids in understanding the spatial distribution of nanoparticles in PDT/PTT treatment across both 2D and 3D models.

CLSM was further used to investigate the intracellular photoactivity of the PNMS and Sto-NFs in Hep G2 cells and their reactive oxygen species (ROS) production for improved photodynamic therapy (PDT) efficacy. The level of ROS generation in cells treated with nanoparticles under 660 nm laser irradiation was monitored using 2′,7′-dichlorodihydrofluorescein diacetate (DCFH-DA) as an intracellular ROS probe. Nonfluorescent DCFH-DA permeates cells, where it is hydrolyzed by cellular esterases to DCFH, which then reacts with ROS to form green fluorescent 2′,7′-dichlorodihydrofluorescein (DCF).^34^ As shown in Figure 3A, cells were incubated with PNMS and Sto-NFs for 24 h and irradiated with or without laser for 10 min. The CLSM images of cells incubated with PNMS exhibited bright green fluorescence, indicating substantial ROS production, which is attributed to higher nanoparticle content in PNMS. In contrast, cells treated with the nontargeting Sto-NFs showed weaker fluorescence and lower ROS levels, which was attributed to the limited cellular internalization. The CLSM images were then analyzed using ImageJ, and the fluorescence intensity of PNMS was significantly higher than that of Sto-NFs (Figure S8). Meanwhile, the cells in both groups consistently revealed a minimal fluorescence without laser irradiation. Next, following the same procedure, cells treated with nanoparticles were stained with calcein-AM and propidium iodide (PI) for a live/dead assay. In Figure S9, all cells were alive without laser irradiation, showing the good biocompatibility of these nanoparticles. After laser irradiation for 10 min, PNMS-treated cells showed significantly higher cell death compared to Sto-NFs treated cells. The live/dead analysis highlighted the strong cell-killing effect of the targeted PNMS (Figure 3B).

**Figure 3:**
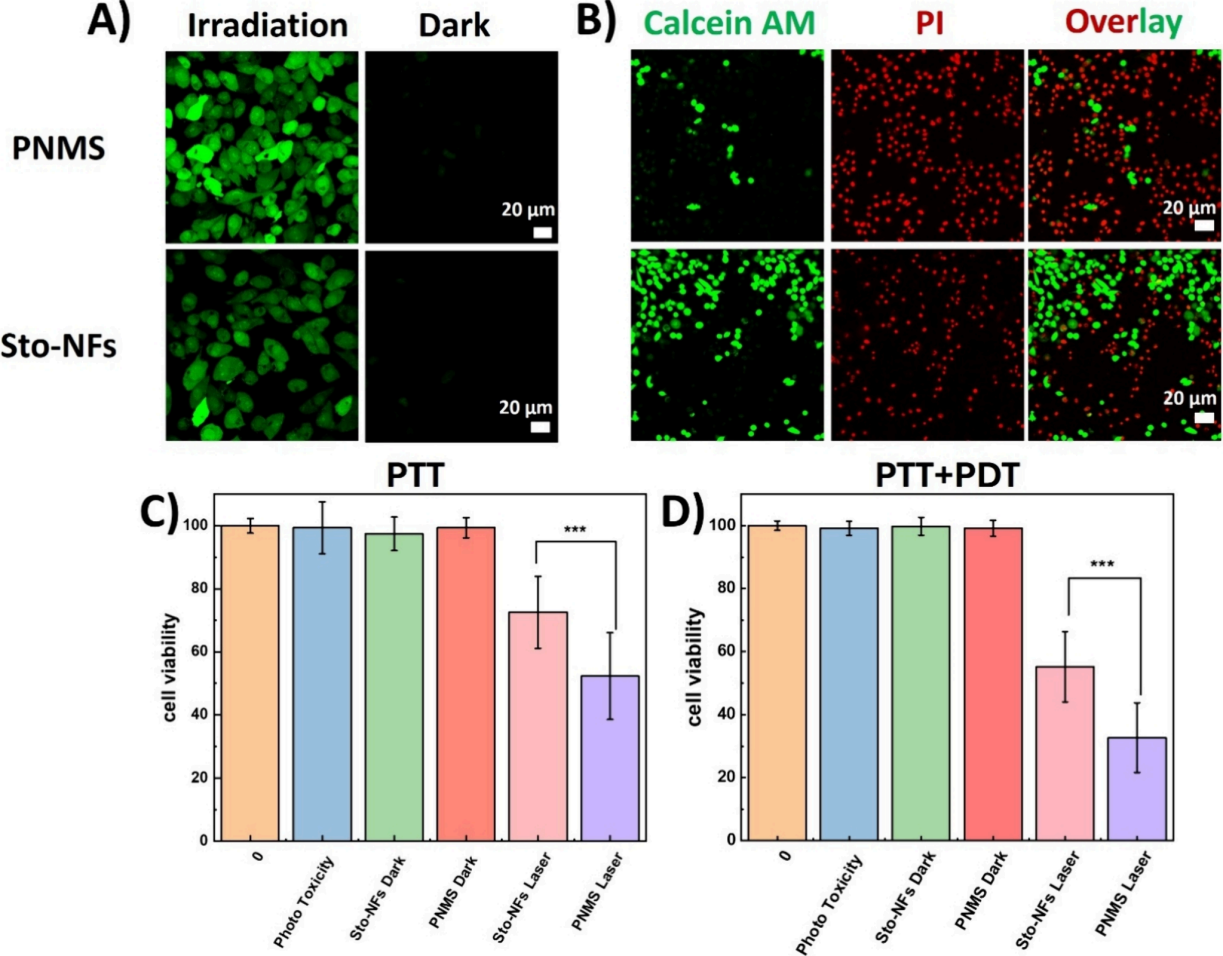
(A) CLSM images of intracellular ^1^O_2_ generation in Hep G2 cells measured by DCFH-DA after treatment of the cells with PNMS and Sto-NFs with and without 660 nm laser irradiation (0.2 W/cm^2^, 10 min), Scale bar = 20 μm. (B) CLSM images of Hep G2 cells stained with calcein-AM/PI, incubated with PNMS and Sto-NFs with 660 nm laser irradiation (0.2 W/cm^2^, 10 min), Scale bar = 20 μm. (C) PTT and (D) PTT+PDT therapy efficacies, dark cytotoxicity, and phototoxicity of Hep G2 cells incubated with PNMS or Sto-NFs, determined by a CCK 8 assay. 0 refers to cells without treatment, phototoxicity refers to cells that are irradiated in the absence of nanoparticles.

PDT-induced cytotoxicity is mainly driven by the generation of short-lived ROS, with the photosensitizer’s chemical structure, subcellular localization and light delivery parameters determining the dominant cell death mechanisms.^35,36^ In contrast, PTT-induced cytotoxicity relies on heat-induced protein denaturation, where higher temperatures lead to faster and more effective cell killing.^7^ Based on the photothermal property of these nanoparticles (Figure 1D,E), we evaluated the effects of photothermal therapy (PTT) and combined PDT/PTT on cells. To assess the therapeutic efficacy of PTT, cells were incubated with PNMS and Sto-NFs for 24 h, irradiated with a 660 nm laser for 10 min, and then cell viability was measured directly by a Cell Counting Kit-8 (CCK 8) assay, as PTT has an immediate effect on cell viability. PTT-induced cell killing is shown in Figure 3C, with PNMS showing significantly higher efficiency compared to Sto-NFs. Subsequently, following the same procedure, cells were incubated overnight before cell viability was measured using the CCK 8 assay to assay the combined PDT/PTT effect, because PDT affects cells in a slower manner. As shown in Figure 3D, the combined PDT/PTT therapy proved more effective than PTT alone, with overall high cell-killing efficiency.

These results indicate that, compared to Sto-NFs, the targeting ability of PNMS significantly increased cellular internalization, leading to greater accumulation of the nanoparticles and photosensitizers inside the cells. This enhances the overall efficacy of the combined PDT/PTT therapy.

Motivated by the aforementioned results, we extended the evaluation of therapeutic efficacy to 3D models as a mimic of tumor tissue. Multicellular spheroids, with diameters of 400–500 μm, were constructed to evaluate the targeting efficiency, penetration capability, and tumor-killing capacity of PNMS and Sto-NFs in Hep G2 spheroids under PTT and combined PDT/PTT therapy. Multicellular spheroids replicate several features of solid tumors, including complex multicellular architecture, mass transport limitations, and pathophysiological gradients.^37,39^ First, the spheroids were incubated with nanoparticles for 6 h. As shown in Figure 4A, CLSM images revealed the distinctive distribution and accumulation of nanoparticles within the spheroids. The nanofibers exhibited strong red fluorescence, demonstrating effective imaging capacity. Spheroids incubated with PNMS exhibited significantly higher red signals at the periphery compared to those treated with Sto-NFs, consistent with the line fluorescence intensity analysis. The targeted PNMS were primarily concentrated in the peripheral areas and only marginally penetrated into the deep areas of spheroids in 6 h. Next, the Hep G2 spheroids were incubated with PNMS and Sto-NFs for 24 h and further evaluated for particle uptake and penetration. At the cross sections, both nanoparticles accumulated and penetrated into the spheroids, with fluorescence detectable even in the center of the tumor spheroids. In contrast, blank spheroids showed limited red signals (Figure S10). Furthermore, the overall diameter of cell spheroids typically ranges from 50 to 800 μm, while the individual cells comprising these spheroids generally measure around 15–17 μm in diameter. This suggests that the intercellular spacing within the spheroids can span several micrometers, depending on the cell type and the degree of compaction within the spheroidal structure.^38^ Larger 3D spheroids, such as those used in this study (approximately 500–600 μm), tend to have even larger gaps due to factors such as nutrient and oxygen gradients, extracellular matrix (ECM) accumulation, cell death, and mechanical stress.^40^ This structural feature supports the observation that PNMS and Sto-NFs effectively penetrated the entire spheroid. Moreover, PNMS exhibited a stronger red signal compared to Sto-NFs in the spheroid, indicating higher accumulation, which is consistent with the line fluorescence intensity analysis (Figure 4B). These results demonstrate that mannose glycopolymer-conjugated nanoparticles not only exhibit selective tumor-targeting ability but also achieve effective penetration in 3D tumor models.

**Figure 4:**
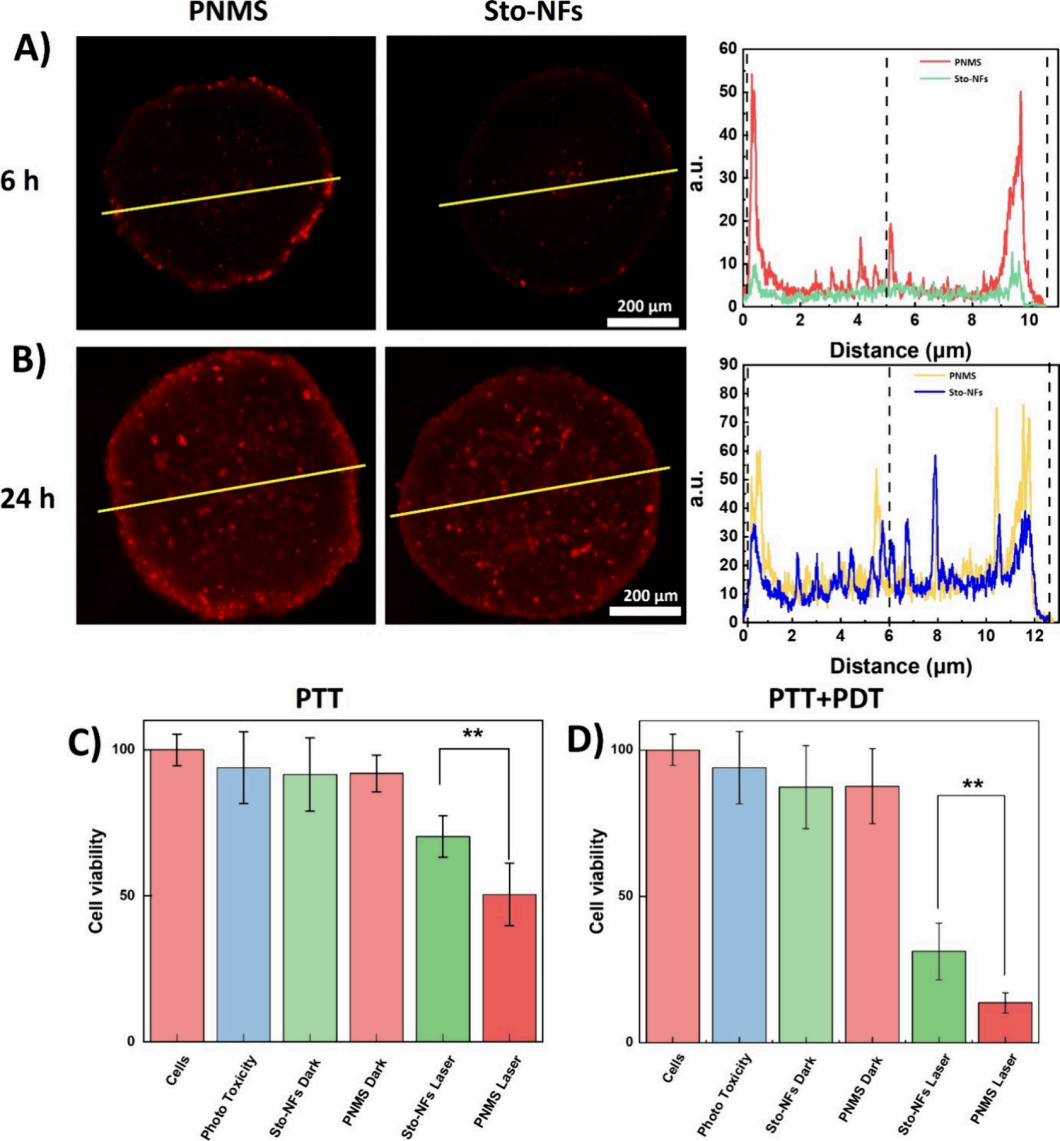
CLSM penetration images of spheroids of PNMS and Sto-NFs into Hep G2 cells, and line fluorescence intensity analysis of spheroids (A) after 6 h and (B) after 24 h, scale bar = 200 μm. (C) PTT and (D) PTT+PDT therapeutic efficacies, dark cytotoxicity and phototoxicity in Hep G2 spheroids with PNMS and Sto-NFs determined by cytotoxicity assays.

After confirming tissue penetration, PTT and combined PDT/PTT treatments were conducted on the spheroids. Prior to treatment, phototoxicity and dark toxicity assays were performed, confirming that these nanoparticles had no cell killing effects without light irradiation (Figure 4C,D). We first evaluated the PTT efficiency of PNMS and Sto-NFs. After 24 h of incubation, spheroids were irradiated with a 660 nm laser (0.2 W/cm^–2^) for 10 min, and then a cytotoxicity assay was performed. As shown in Figure 4C, spheroids treated with Sto-NFs showed 30% cell killing, while those treated with PNMS showed 50%, demonstrating the benefit of the active targeting mediated by mannose glycopolymer. Subsequently, combined PDT/PTT treatment was performed. As shown in Figure 4D, PNMS demonstrated significantly higher efficacy, with approximately 87% cell killing, which can be attributed to its targeting function and is consistent with its enhanced tissue penetration and accumulation. In contrast, Sto-NFs showed approximately 70% cell killing, still demonstrating high therapeutic capacity. Compared to the nanofibers alone, which achieved approximately 80% cell killing,^12^ our platform exhibited a higher cytotoxicity of 87% even at a lower nanofiber concentration. This enhancement is attributed to the increased local concentration of nanofibers confined within the cavity of the stomatocytes. These results confirm that PNMS exhibits a high therapeutic efficacy under combined PDT/PTT treatment, highlighting the advantages of its targeting ability.

## Conclusions

We developed a dual-nanoparticle platform for combined photodynamic therapy (PDT) and photothermal therapy (PTT), in which nanofibers of peptide-porphyrin conjugates were encapsulated in the nanocavity of mannosylated stomatocytes (PNMS). This approach allows to integrate different features into one system without the need to compromise in the design of the individual particles. PNMS exhibit enhanced tumor imaging, improved targeting, and increased cellular uptake in tumor cells and 3D tumor spheroids. It demonstrates excellent photothermal and photodynamic performance, characterized by significant reactive oxygen species (ROS) generation and temperature elevation under laser irradiation. These findings highlight the potential of this nanomedicine platform as a promising strategy for precision cancer therapy, facilitating targeted drug delivery and providing a versatile approach to enhance treatment efficacy in tumor microenvironments.

## Supplementary Material



## Notes

Materials and methods; scanning electron microscope image of stomatocytes; size distribution of PNMS, Sto-NFs, and stomatocytes in PBS; photothermal images of empty stomatocytes upon irradiation with a 660 nm laser; photothermal images of water upon irradiation with a 660 nm laser at a power density of 1.5 W; surface plasmon resonance (SPR) analysis illustrating the binding of stomatocytes with MBL; time-dependent localization of nanoparticles in lysosomes evaluated at 8, 12, 18 h; CLSM images of Hep G2 cells incubated with PNMS after 24 h; DCFH fluorescence intensity profiles of PNMS and Sto-NFs; CLSM images of Hep G2 cells stained with calcein-AM/PI, incubated with PNMS, Sto-NFs without 660 nm laser irradiation; CLSM images of blank Hep G2 spheroids; evaluation of loading efficiency of nanofibers into stomatocytes, compared to empty stomatocytes; and kinetic values (ka, kd, KD, Rmax) obtained from fitting experimental SPR curves with a 1:1 Langmuir binding model (PDF)
