# Supplementary material for: Targeted Peptide Nanofiber-Loaded Stomatocytes for Combined Photodynamic and Photothermal Therapy
Source: ACS Appl Mater Interfaces. 2025 Jun 3;17(24):35230–9. doi: 10.1021/acsami.5c06448 (PMC12186217; doi:10.1021/acsami.5c06448)
Supplement: Supplementary file 1 [file am5c06448_si_001.pdf]

Supporting Information

Targeted peptide nanofiber-loaded stomatocytes for combined photodynamic and photothermal therapy

Yuechi Liu [a], Duc H.T. Le [a], Gokhan Yilmaz [b], Lars Paffen [a], Shukun Li [a], Alexander B. Cook [a], Tania Patino Padial [a], Loai Abdelmohsen [a], C. Remzi Becer [b], Bingbing Sun [a], Jan C.M. van Hest* [a]*

a Y. Liu, Dr. D. H. T. Le, Dr. L. Paffen, Dr. S. Li, Dr. A. B. Cook, L. Dr. T. Patino Padial, Dr. L. Abdelmohsen, Dr. B. Sun, Prof. J. C. M. van Hest

Bio-Organic Chemistry, Institute of Complex Molecular Systems

Eindhoven University of Technology

Helix, P. O. Box 513, 5600 MB, Eindhoven (The Netherlands)

J.C.M.v.Hest@tue.nl

b.sun@tue.nl

b G. Yilmaz, C. R. Becer.

Department of Chemistry

University of Warwick

Coventry, CV4 7AL, UK.

Experimental Procedures

1. Materials and Methods

1.1 General method for stomatocyte preparation
1.2 Preparation of azide-functional stomatocytes
1.3 CuAAC of alkyne-mannose glycopolymer with N_3-stomatocytes
1.4 Self-assembly of $PHHPEG_6$
1.5 $PHHPEG_6$ nanofibers encapsulation in stomatocytes
1.6 $PHHPEG_6$ nanofiber loading efficiency
1.7 Singlet oxygen generation of PNMS and Sto-NFs in solution
1.8 Temperature changes during photo-irradiation
1.9 SPR measurements
2.0 Singlet oxygen generation in vitro
2.1 Cytotoxicity studies (CCK8) *in vitro*
2.2 Flow cytometry
2.3 Cellular uptake of PNMS and Sto-NFs
2.4 Formation of spheroids and tissue penetration of nanoparticles
2.5 Cytotoxicity assay in spheroid models
2.6 Statistical analysis

2. Supplementary figures

FigureS1.Scanning electron microscope image of stomatocytes
FigureS2.Size distribution of PNMS, Sto-NFs, and stomatocytes in PBS at pH 5.0, 6.5, and 7.4, as well as after overnight incubation, measured by DLS.
FigureS3.Photothermal images of empty stomatocytes upon irradiation with a 660 nm laser at a power density of 1.5 W.

FigureS4.Photothermal images of water upon irradiation with a 660 nm laser at a power density of 1.5 W.
FigureS5.Surface plasmon resonance (SPR) analysis illustrating the binding of stomatocytes with MBL.
FigureS6.Time-dependent localization of nanoparticles in lysosomes evaluated at 8,12,18 h.
FigureS7.CLSM images of Hep G2 cells incubated with PNMS after 24 h, the stomatocytes were stained with FITC, the nucleus was stained with Hoechst.
FigureS8.DCFH fluorescence intensity profiles of PNMS and Sto-NFs in Figure 3A irradiation, by Image J analysis.
FigureS9.CLSM images of Hep G2 cells stained with calcein-AM/PI, incubated with PNMS, Sto-NFs without 660 nm laser irradiation.
FigureS10.CLSM images of blank Hep G2 spheroids.
TableS1.Evaluation of loading efficiency of nanofibers into stomatocytes, compared to empty stomatocytes.
TableS2.Kinetic values (ka, kd, KD, Rmax) obtained from fitting experimental SPR curves with a 1:1 Langmuir binding model.

3. References

All polymers and peptides were synthesized according to procedures previously published.

1.1 General method for stomatocyte preparation

Stomatocytes were prepared following a previous published procedure.[1, 2] In a 15 mL vial, PEG_{22}-$PDLLA_{95}$ and PEG_{44}-$PDLLA_{95}$ block copolymers (1:1 w/w, 20mg) were dissolved in 2 mL of THF and dioxane (1:4 v/v) and the vial was sealed with a rubber septum. The solution was stirred at 700 rpm for a minimum of 30 minutes prior to the addition of MilliQ (2 mL, 1 mL h^{-1}) via a syringe pump. A needle was inserted into the septum to release pressure. The resulting cloudy suspension was transferred into a prehydrated dialysis bag (SpectraPor, MWCO: 12-14 kDa Mw). Dialysis was performed against a pre-cooled salt solution (usually 50 mM NaCl) at 4 °C for a maximum of 24 hours, with a water change after the first hour.

1.2 Preparation of azide-functional stomatocytes

In a 15 mL vial, PEG_{44}-$PDLLA_{95}$, PEG_{22}-$PDLLA_{95}$, and N_3-PEG_{67}-$PDLLA_{70}$ block copolymers (in a ratio of 10:7:3 w/w/w, totaling 20 mg) were dissolved in 2 mL of a THF and dioxane mixture (1:4 v/v). The vial was sealed with a rubber septum and the solution was stirred at 700 rpm for a minimum of 30 minutes. Milli-Q water (2 mL) was then added via a syringe pump at a rate of 1 mL/h, while a needle was inserted into the septum to release pressure. The resulting cloudy suspension was transferred into a prehydrated dialysis bag (SpectraPor, MWCO: 12-14 kDa, 2 mL/cm). Dialysis was performed against 75 mM NaCl at room temperature for a maximum of 24 hours, with a water change after the first hour.

1.3 CuAAC of alkyne-mannose glycopolymer with N_3-stomatocytes

Alkyne mannose glycopolymer and N_3-stomatocytes were reacted in pure water at a molar ratio of 1.2:1, the concentration of the alkyne mannose glycopolymer was 0.76 mM and 0.64 mM for the azide groups on the stomatocytes. $CuSO_4$, THPTA, and sodium ascorbate were dissolved in pure water at a molar ratio of 1:5:5, with the [$CuSO_4$] 1.27 mM, [THPTA] 6.39 mM and [sodium ascorbate] 6.39 mM. Then the reagents were added to the polymer mixture, to a total volume of 500 μL and the reaction proceeded at room temperature overnight (at least 12 h). After the click reaction, the product was first transferred to a 1 mL Eppendorf tube. MilliQ water was added to the mixture, which was then centrifuged at 12,000 rpm for 4 minutes. The

supernatant was removed, 1 mL of MilliQ water was added, and this procedure was repeated three times.

1.4 Self-assembly of PHHPEG$_6$

The PHHPEG$_6$ powder was dissolved in DMSO at a concentration of 100 mg/mL. Then, 1 mL MilliQ water was added into 10 μL of PHHPEG$_6$ solution under ultrasonication for 30 s to form PHHPEG$_6$ nanofibers, which were aged one day before use.

1.5 PHHPEG$_6$ nanofibers encapsulation in stomatocytes

To encapsulate PHHPEG$_6$ nanofibers in the stomatocyte cavity, 500 μL of 20 mg/mL stomatocytes were stirred with 1 mL 1 mg/mL PHHPEG$_6$ nanofibers for 24 h at a speed of 100 rpm under 4 °C. After overnight stirring, the mixture was transferred to a 2 mL Eppendorf tube and then centrifuged at 12,000 rpm for 4 minutes. The supernatant was removed, 2 mL of MilliQ water was added, and this procedure was repeated three times. The nanofibers-loaded stomatocytes were then suspended in 500 μL of pH 6.5 PBS for 30 minutes at room temperature during centrifugation with a speed of 1,000 rpm. Finally, the mixture was washed with MilliQ water following the above procedure, which was repeated three times. The nano-fiber loaded stomatocytes (Sto-NFs) were suspended in 1 mL of Milli-Q water.

1.6 PHHPEG$_6$ nanofiber loading efficiency

A mixture of Milli-Q water and DMSO was prepared in a volume ratio of 1:4. The nanofibers at a concentration of 1 mg/mL were then diluted 2, 5, 10, 20, 50, and 100 times to create a standard curve. For measurement, 20 μL of the nanofibers-loaded samples were dissolved in 80 μL of DMSO, and the blank sample (empty stomatocytes) was similarly dissolved in DMSO. The fluorescence was measured at an excitation wavelength of 518 nm and an emission wavelength of 660 nm.

1.7 Singlet oxygen generation of PNMS and Sto-NFs in solution

The singlet oxygen probe SOSG was added to PNMS, Sto-NFs, empty stomatocytes and water at a final concentration of 20 μM, the particles concentration were at concentration 800 μg/mL. All sample solutions were then illuminated with a 660 nm laser (BeamQ Lasers) at a photodensity of 0.2 W/cm^2 for 10 min. The SOSG endoperoxide (SOSG-EP) was determined by measuring the fluorescence intensity at 540 nm as a function of time by a Spark 10 M microplate reader (TECAN, Switzerland). Milli-Q water and empty stomatocytes under light illumination were set as the negative controls.

1.8 Temperature changes during photo-irradiation

PNMS, empty stomatocytes and Milli-Q water solutions were photoirradiated by a 660 nm laser light (0.2 W/cm^2) for 10 min, the particles concentration were at concentration 800 μg/mL. Temperature profiles of the solutions (500 μL in a 1.5 mL Eppendorf tube) during irradiation were measured and recorded by a C.A 1821 thermocouple thermometer (Chauvin Arnoux).

1.9 SPR measurements

Interaction analysis between mannose binding lectin (MBL) and stomatocytes was conducted using surface plasma resonance (SPR) on a BIAcore T200 system (GE Healthcare). MBL (0.005 mg/mL) was immobilized via a standard amino-coupling protocol onto a CM5 sensor chip that was activated by flowing a 1/1 mixture of 0.1 M N-hydroxysuccinimide and 0.05 M N-ethyl-N-(dimethylaminopropyl)- carbodiimide over the chip for 5 min at 25 °C at a flow rate of 5 mL/min. Subsequently, all channels were blocked with ethanolamine (1 M pH 8.5) for 10 min at 5 μL/min to remove remaining reactive groups. All experiments were conducted with

HEPES-buffered saline (HBS) (0.10 M HEPES, 0.9 M NaCl, 1 mM $CaCl_2$, adjusted to pH 7.4) and filtered using a 0.2 μm regenerated cellulose syringe filter. Glycopolymer concentrations (0.5−8 μM) per stomatocyte sample were calculated on average. Sensorgrams for each concentration were recorded using 300 s (on period), followed by 200 s of buffer only (off period). Regeneration of the sensor chip surfaces was performed using 10 mM HEPES (pH 7.4), 150 mM NaCl, 10 mM EDTA, and 0.01% TWEEN 20 surfactant solution. Kinetic data were evaluated using a single set of sites (1/1 Langmuir binding) model, by using the BIAevaluation 3.1 software.

2.0 Singlet oxygen generation in vitro

Hep G2 cells were seeded in μ-Slide 8 Wells (5×10^4 cells/well). After culture for 24 h, the cells were incubated with PNMS and Sto-NFs (800 μg/mL). Cell culture medium was removed after 24 h incubation and washed three times with PBS. The cells were then incubated with the fluorescent probe DCFHDA (20 μM) for 30 min at 37 °C in the dark, washed three times with PBS, and resuspended in 200 μL of PBS. The cells were subsequenlty treated with a 660 nm laser for 10 min at a photodensity of 0.2 W/cm^2 and examined using CLSM.

2.1 Cytotoxicity studies (CCK8) in vitro

Hep G2 cells were seeded in 96-well tissue culture plates (5×10^3 cells/well). After culture for 24 h they were incubated with PNMS and Sto-NFs at concentration 800 μg/mL. After 24 h incubation, the cell culture medium was removed, the cells were washed three times with PBS and resuspended in 100 μL of DMEM. For PDT/PTT therapy, the cells were then treated with a 660 nm laser at 0.2 W/cm^2 for 10 min and continually cultured for an additional 24 h. The cell viability was then analyzed by the CCK-8 assay. For PTT therapy, the cells were treated with a 660 nm laser for 10 min and directly analyzed by the CCK-8 assay. Hep G2 cells treated with PNMS and Sto-NFs without light illumination were set as the control.

2.2 Flow cytometry

Flow cytometry analysis was conducted by measuring the red fluorescence originating from the nanoparticles with a FACS Canto II (BD Biosciences). Data obtained was further processed using FlowJo v10.9.0 (Tree Star, Inc.) to calculate the mean fluorescence intensity (MFI; geometric mean) and the frequencies of red fluorescence -positive populations. The orange peak in Figure 2D corresponds to the blank (untreated) cells, which serve as the negative control group. It is a baseline for comparison with treated samples.

2.3 Cellular uptake of PNMS and Sto-NFs

Hep G2 cells were seeded in μ-Slide 8 Wells (5×10^4 cells/well). After culturing for 24 h, the cells were incubated with PNMS and Sto-NFs (800 μg/mL) for 6 h and 24 h, and then washed three times with PBS. Subsequently, the cells were stained with Wheat germ agglutinin (WGA-488) for 5 min, LysoTracker Green, endoplasmic reticulum (ER)-Tracker green for 30 min and Hoechst 33342 (10 μg/mL) for 10 min at 37°C. Then, the cells were washed three times with PBS, resuspended in 200 μL of PBS, and immediately analyzed with CLSM. Flow cytometry was performed on a FACS Aria III (BD Biosciences) equipped with a 70 μm nozzle. Events representing single cells were gated based on the forward height scatter vs the forward area scatter. For each measurement, fluorescence intensities of 10000 individual cells were recorded and analyzed using FlowJo software. The fluorescence of PNMS and Sto-NFs (800 μg/mL) in Hep G2 cells, localized in lysosomes at 8, 12, and 18 hours, was measured by CLSM.

2.4 Formation of spheroids and tissue penetration of nanoparticles

A petri dish for preparing spheroids was coated with 0.75% (w/v) agarose gels. Suspensions (1×10^4 cells per 30 uL drop) of Hep G2 cells were added and cultured on the prepared agarose gel. A single spheroid, with an average diameter of 400-500 μm, was obtained due to aggregation of Hep G2 cells after three days. The spheroids were washed twice using PBS and incubated later with fresh DMEM medium containing the PNMS and Sto-NFs (1.5 mg/mL). Three-hour post-incubation, clearance of spheroids was carried out at 37 ºC for CLSM imaging as previously published.[3] First, spheroids were washed with pre-heated PBS (37 ºC) and fixed in paraformaldehyde (Sigma) (4% (v/v) in PBS) for 30 min at 37 ºC. Subsequently, fixed spheroids were embedded in collagen (Themo Fisher Scientific) gels in each well from an ibidi chamber. The spheroids were then cleared by incubating in fructose (Sigma-Aldrich) solutions containing 0.5% (v/v) 1-thiolglycerol (Sigma-Aldrich) with increasing fructose concentrations, i.e., 28.75%, 57.5%, and 115% (w/v) at 37 ºC. Spheroids were imaged using the SP8 confocal microscope. Images and fluorescence intensity distributions were processed using FIJI (http://fiji.sc/).

2.5 Cytotoxicity assay in spheroid models

Spheroids were prepared in a 96-well plate as aforementioned. Spheroids were washed and then incubated with fresh media containing PNMS and Sto-NFs (1.5 mg/mL) for 24 hours followed by irradiation (660 nm, 0.12 W/cm^2, 10 min). To determine cell viability, the ViaLight™ Plus Cell Proliferation and Cytotoxicity BioAssay Kit (LONZA) were used. Spheroids were completely lysed using the kit lysis buffer to release intracellular adenosine triphosphate (ATP) from alive cells. Reagents for ATP assays comprising of the substrates and luciferase were added to the cell lysate solution and the bioluminescence was determined. Relative cell viability was calculated by the ratio of alive cells in PDT/PTT or only PTT treated spheroids vs. alive cells from spheroids without treatment in the same plate. Data is shown as the average standard deviation from three spheroids treated in the same condition (n = 3).

2.6 Statistical analysis

Data are expressed as mean ± standard deviation.

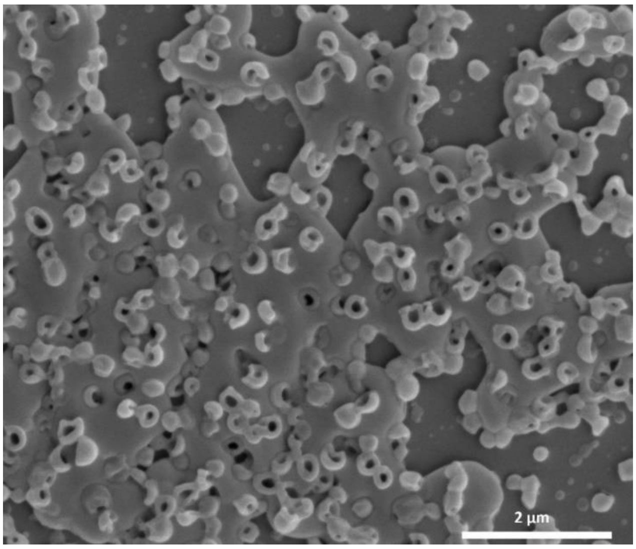

Figure S1. Scanning electron microscope image of stomatocytes, scale bar 2 μm.

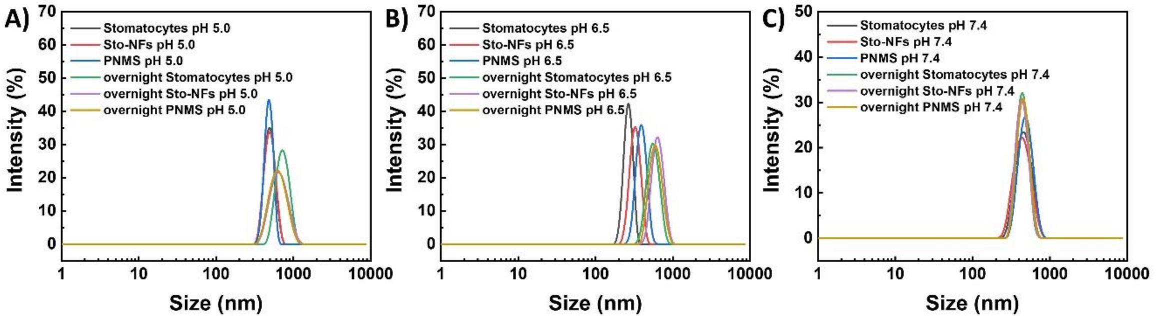

Figure S2. Size distribution of PNMS, Sto-NFs, and stomatocytes in PBS at A) pH 5.0, B) 6.5, and C) 7.4, as well as after overnight incubation, measured by DLS.

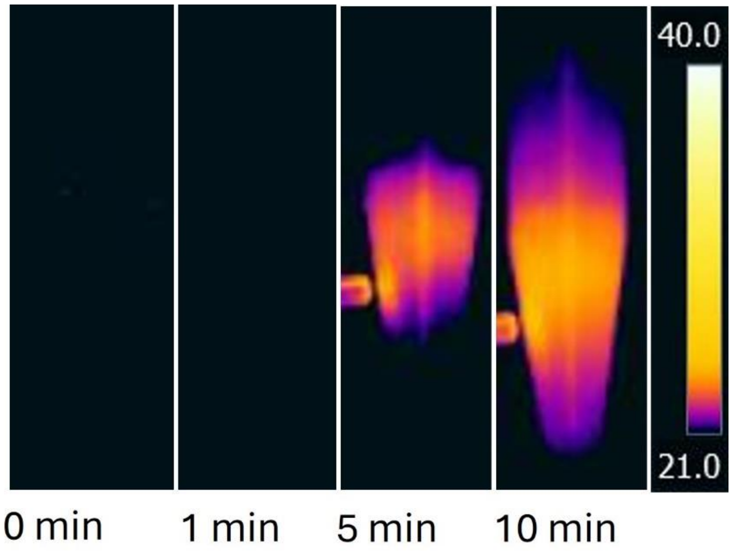

Figure S3. Photothermal images of empty stomatocytes upon irradiation with a 660 nm laser at a power density of 1.5 W.

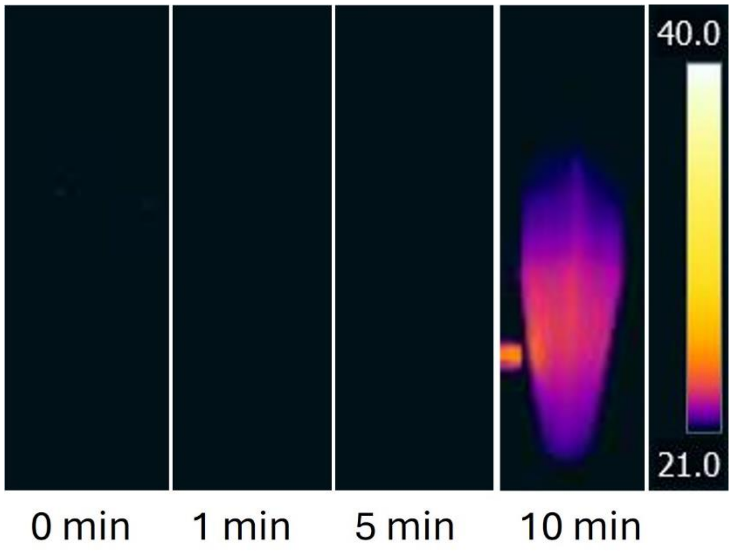

Figure S4. Photothermal images of water upon irradiation with a 660 nm laser at a power density of 1.5 W.

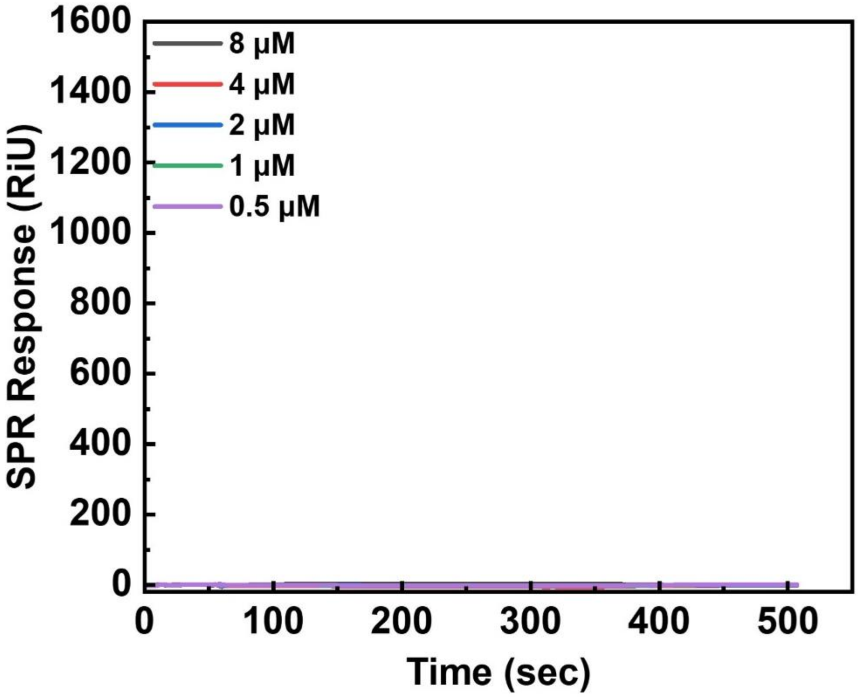

Figure S5. Surface plasmon resonance (SPR) analysis illustrating the binding of stomatocytes with MBL.

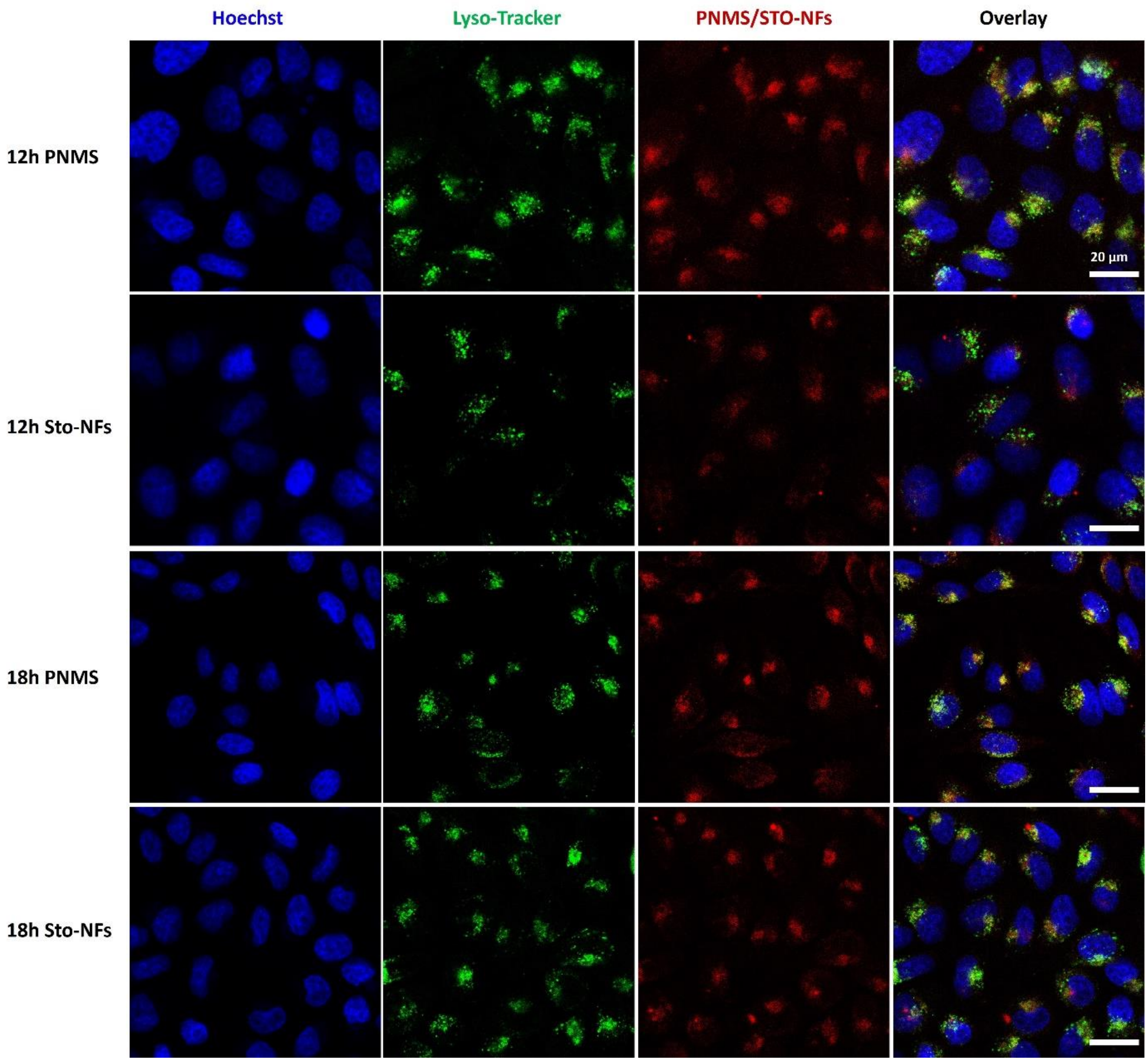

Figure S6. Time-dependent localization of nanoparticles in lysosomes evaluated at 12,18 h. CLSM images indicating the nuclei (stained with Hoechst), lysosomes (LysoTracker Green), and PNMS and Sto-NFs (red), including the overlay. Scale bar = 20 μm.

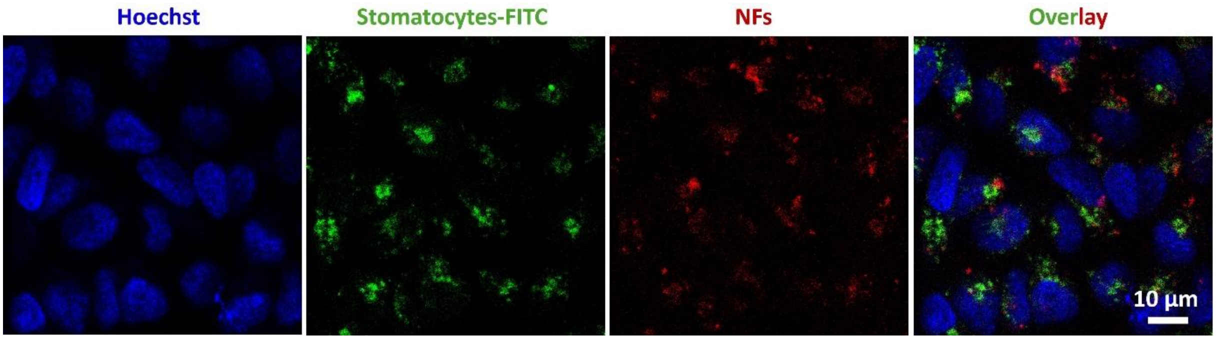

Figure S7. CLSM images of Hep G2 cells incubated with PNMS after 24 h, the stomatocytes were stained with FITC, the nucleus was stained with Hoechst. Scale bar = 10 μm.

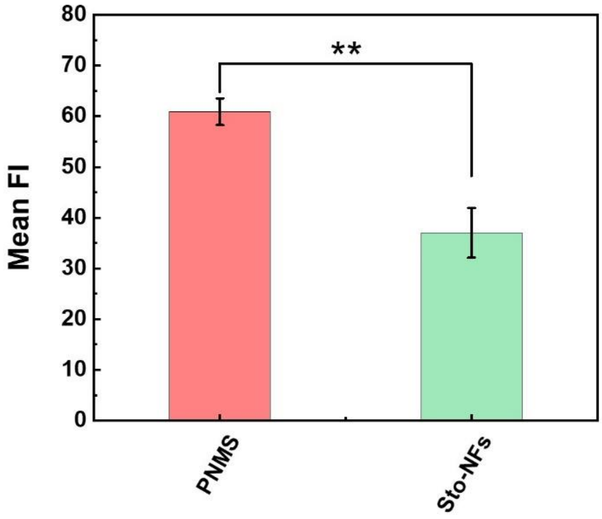

Figure S8. DCFH-DA fluorescence intensity profiles of PNMS and Sto-NFs in Figure 3A, by Image J analysis.

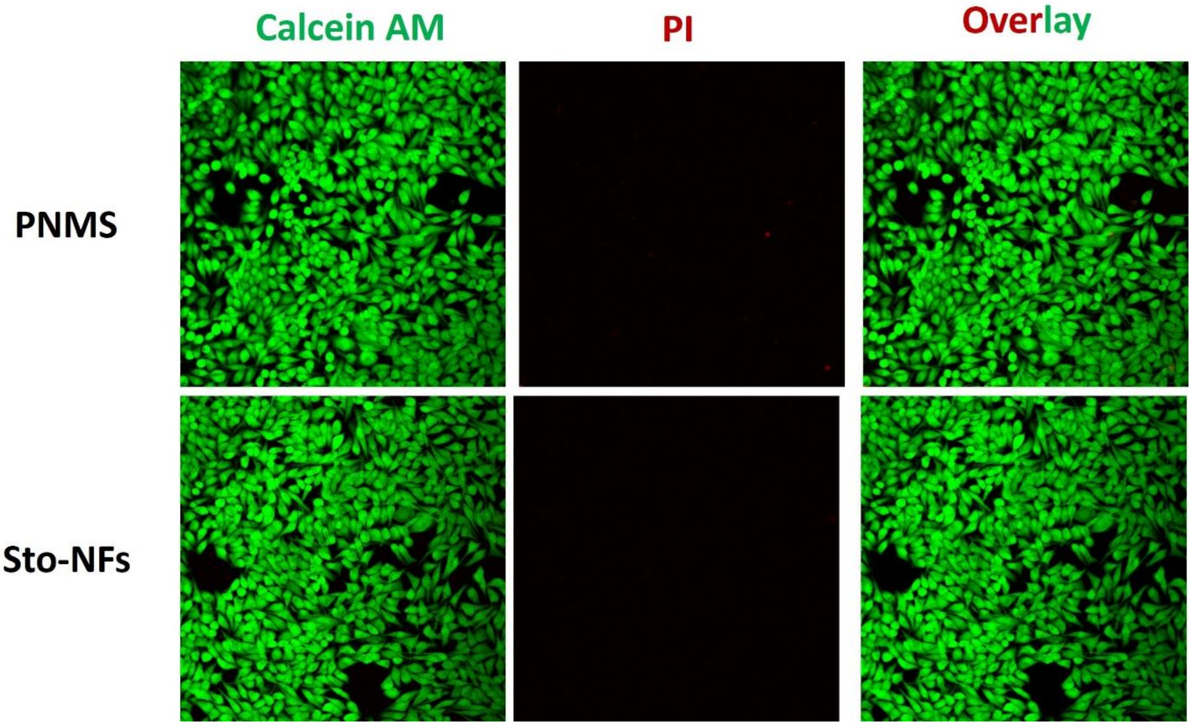

Figure S9. CLSM images of Hep G2 cells stained with calcein-AM/PI, incubated with PNMS, Sto-NFs without 660 nm laser irradiation.

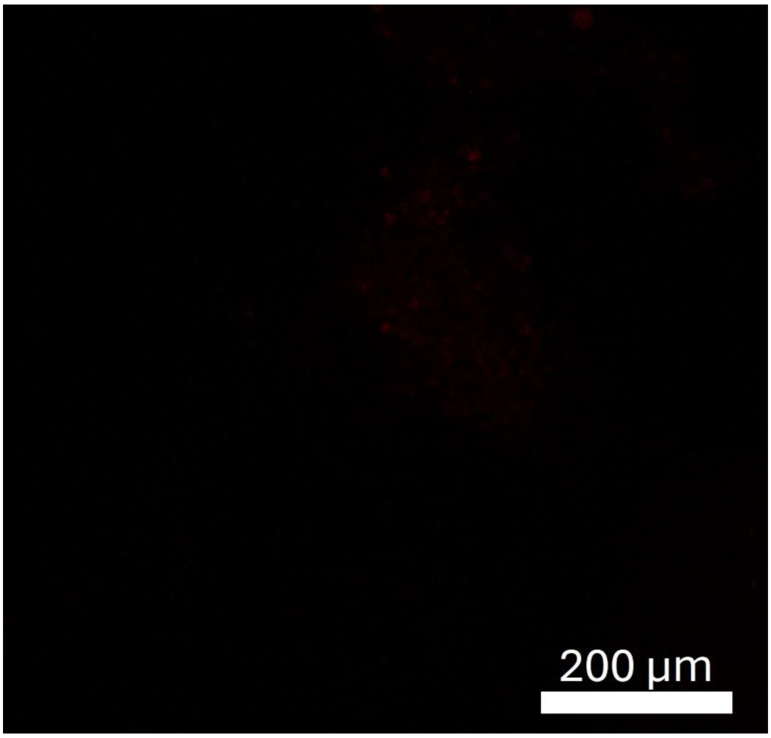

Figure S10. CLSM images of blank Hep G2 spheroids. Scale bar = 200 μm.

Table S1. Evaluation of loading efficiency of nanofibers into stomatocytes, compared to empty stomatocytes.

	Loading efficiency (%)
Loaded stomatocytes	7.70%
Empty stomatocytes	0.00 (The background signal was 12)

Table S2. Kinetic values (k_a, k_d,K_D, R_{max}) obtained from fitting experimental SPR curves with a 1:1 Langmuir binding model.

	$k_a(M^{-1}s^{-1})$	$k_d(s^{-1})$	$K_D(M)$	$R_{max}(RU)$
Man-stomatocytes	629	1.05*10-6	1.67*10-9	1180
PNMS	674	5.56*10-6	8.25*10-9	1200

3. References

(1) Abdelmohsen, L. K.; Nijemeisland, M.; Pawar, G. M.; Janssen, G. J.; Nolte, R. J.; van Hest, J. C.; Wilson, D. A. Dynamic Loading and Unloading of Proteins in Polymeric Stomatocytes: Formation of an Enzyme-Loaded Supramolecular Nanomotor. *ACS Nano.* **2016**, *10 (2)*, 2652-2660.

(2) Abdelmohsen, L. K. E. A.; Williams, D. S.; Pille, J.; Ozel, S. G.; Rikken, R. S. M.; Wilson, D. A.; van Hest, J. C. M. Formation of Well-Defined, Functional Nanotubes via Osmotically Induced Shape Transformation of Biodegradable Polymersomes. *J. Am. Chem. Soc.* **2016**, *138 (30)*, 9353-9356.
(3) van Oppen, L.; Pille, J.; Stuut, C.; van Stevendaal, M.; van der Vorm, L. N.; Smeitink, J. A. M.; Koopman, W. J. H.; Willems, P.; van Hest, J. C. M.; Brock, R. Octa-arginine Boosts the Penetration of Elastin-Like Polypeptide Nanoparticles in 3D Cancer Models. *Eur. J. Pharm. Biopharm.* **2019**, *137*, 175-184.
